# First-Principles Study of Atomic Diffusion by Vacancy Defect of the L1_2_-Al_3_M (M = Sc, Zr, Er, Y) Phase

**DOI:** 10.3390/molecules28186727

**Published:** 2023-09-21

**Authors:** Shuai Liu, Binbin Liao, Baohua Nie, Touwen Fan, Dongchu Chen, Jianglong Zhang, Yu Song

**Affiliations:** 1 School of Materials Science and Hydrogen Energy, Foshan University, Foshan 528000, Chinachendc@fosu.edu.cn (D.C.); 2 Research Institute of Automobile Parts Technology, Hunan Institute of Technology, Hengyang 421002, China; 3 Shenyuan Honors of College, Beihang University, Beijing 100191, China; 4 Shenzhen Rspower Technology Co., Ltd., Shenzhen 518000, China

**Keywords:** first-principles calculation, vacancy defect, atomic diffusion, L1_2_-Al_3_M

## Abstract

Atomic diffusion by the vacancy defect of L1_2_-Al_3_M (M = Sc, Zr, Er, Y) was investigated based on a first-principles calculation. The point defect formation energies were firstly evaluated. Then, the migration energy for different diffusion paths was obtained by the climbing-image nudged elastic band (CI-NEB) method. The results showed that Al atomic and M atomic diffusions through nearest-neighbor jump (NNJ) mediated by Al vacancy (V_Al_) were, respectively, the preferred diffusion paths in Al_3_M phases under both Al-rich and M-rich conditions. The other mechanisms, such as six-jump cycle (6JC) and next-nearest-neighbor jump (NNNJ), were energetically inhibited. The order of activation barriers for NNJ(Al-V_Al_) was Al_3_Zr < Al_3_Y < Al_3_Er < Al_3_Sc. The Al_3_Sc phase had high stability with a high self-diffusion activation barrier, while the Al_3_Zr and Al_3_Y phases were relatively unstable with a low self-diffusion activation energy. Moreover, the atomic-diffusion behavior between the core and shell layers of L1_2_-Al_3_M was also further investigated. Zr atoms were prone to diffusion into the Al_3_Y core layer, resulting in no stable core-shelled Al_3_(Y,Zr), which well agreed with experimental observation.

## 1. Introduction

Aluminum alloys containing transition metals (TM) and rare-earth (RE) elements are widely used in aerospace, high-speed trains, and automobiles [1,2,3]. The formation of L1_2_-Al_3_(RE,TM) nanophases with high thermal stability, such as the core-shelled Al_3_(Sc,Zr) nanophase, can effectively inhibit the recrystallization process of aluminum alloys, achieving high strength toughness and corrosion resistance performance [2]. Based on the high thermostability and coarsening resistance of L1_2_-Al_3_(RE,TM) nanophases, Seidman et al. [4,5,6] developed an Al-RE-TM series of high-temperature aluminum alloys.

Due to the high cost of Sc elements, low-cost elements that can form thermally stable Al_3_M phases were explored to replace Sc elements. A first-principles calculation showed that Er and Yb elements were candidate element to replace Sc elements [7,8]. The Zr element can partially replace Sc element and was considered an ideal element to form the Al_3_Zr shell structure owing to the Al_3_Zr/Al interface with low interface energy and coherent strain energy [9]. The typical core-shelled nanophases, such as Al_3_(Er,Zr) [10] and Al_3_(Yb,Zr) [11], were introduced in aluminum alloys to replace Al_3_(Sc,Zr) nanophases. The formation of these core-shelled Al_3_M phases was explained by the difference in diffusion rates between elements, that is, the rapid diffusion elements were enriched to form a core layer and the slow diffusion elements were segregated to form a shell layer [12]. The core-double-shelled structure that was observed in Al-Sc-Er-Zr alloys contained an Er-enriched core surrounded by a Sc-enriched core and a Zr-enriched outer shell, obtaining a high coarsening resistant and high strength [5,13]. The formation of the core-double-shelled structure well agreed with the prediction by the atomic diffusivity ordering of D_Er_ > D_Sc_ > D_Zr,_

The Y element, as a Sc homologous element with similar physical and chemical properties, was a probable candidate element to replace the Sc element [14]. Zhang et al. [15,16] showed that core-shelled Al_3_(Y,Zr) with a Y-rich core and Zr-rich shell can be formed during the early stage of aging in Al-Y-Zr alloys. The Al_3_Y phase acted as the heterogeneous core to accelerate the precipitation of Al_3_Zr, which well agreed with the atomic-diffusion control mechanism. However, atom probe tomography (APT) showed that Y and Zr atoms were randomly distributed in the L1_2_-Al_3_(Y,Zr), and hybrid structure, rather than core-shelled structure, was observed in the Al_3_(Y,Zr) phase after long-term aging. The strong binding energy between Y and Zr atoms was assumed to explain the formation of hybrid structure Al_3_(Y,Zr) [16]. According to the authors’ previous investigation, the core-shelled Al_3_Y/Al_3_Zr was thermodynamically unstable due to its high coherent strain energy of Al_3_Y/Al_3_Zr. A similar transition from a core-shelled structure to a hybrid structure during long-term aging was also observed in the Al-Yb-Sc alloy [17]. Seidman et al. [17] suggested that the inter-diffusion of Yb and Sc resulted in a uniform distribution of elements throughout the precipitates. Thus, the mechanism of the L1_2_-Al_3_M phase with core-shelled structure or hybrid structure needed to be answered.

Atomic diffusion, especially mediated by vacancy, was very beneficial for understanding the microstructural stability of the L1_2_-Al_3_M phase [18]. Although experimental methods were difficult to investigate atomic diffusion in intermetallic compounds [19,20], first-principles calculations can provide new insights into the microscopic mechanisms of atomic diffusion [18,20]. First-principles calculations by Fan [21,22] showed that with the increase of the atomic number, the diffusion rate of rare-earth elements increased from Sc to Y, La, and then decreased to Lu. Shi et al. [23] investigated the atomic diffusion of pure and transition-element (TM)-doped L1_2_-Al_3_Sc based on first principles and found that under a strong Al-rich condition, the V_Sc_ defect obtained low formation energy and the NNJ mechanism mediated by V_Al_ was most favorable for Sc atomic diffusion. TM dopants increased diffusion activation energy for dominant Al_3_Sc diffusion. However, the atomic diffusion between the core layer and the shell layer for the core-shelled L1_2_-Al_3_M phase was far from fully understood.

The atomic-diffusion mechanism in the L1_2_-Al_3_M (M = Sc, Er, Y, Zr) phases was investigated based on the first-principles in the present work. The formation energy of point defects was calculated. Then, migration energy along possible diffusion was analyzed by the climbing-image nudged elastic band (CI-NEB) method [24]. Thus, the diffusion activation energy was obtained. Furthermore, the atomic-diffusion behavior between the core and shell layers for the core-shelled L1_2_-Al_3_M phase was also further illustrated. The purpose of this study was to reveal the microscopic mechanism of atomic diffusion in the L1_2_-Al_3_M phases and core-shelled L1_2_-Al_3_M phase, providing a theoretical guidance for the development of high-performance aluminum alloys containing TM and RE elements.

## 2. Results and Discussion

### 2.1. Defect Formation Energy

In order to evaluate the difficulty of point defect formation, the formation energy of point defects in Al_3_M was calculated as [25]

(1) $$E_{f}^{def}=E_{total}^{def}-E_{total}^{bulk}-\sum_{i}\Delta n_{i}\mu_{i}$$

Here, $E_{total}^{def}$ and $E_{total}^{bulk}$ are the total energy of the defective supercell and the total energy of the defect-free supercell, respectively. n_i_ represents the number of i atoms (i = Al or M) that increased ($\Delta n_{i}>0$) or decreased ($\Delta n_{i}<0$) when defects were formed, and *μ*_i_ is the relative chemical potential of i atoms.

To maintain a stably balanced L1_2_ phase, its chemical potential should meet the following requirement:

(2) $$3\Delta \mu_{Al}+\Delta \mu_{M}=\Delta H_{f}^{Al_{3}M}$$

Here, $\Delta H_{f}^{Al_{3}M}$ is the formation enthalpy of the unit chemical formula Al_3_M in the solid state; $\Delta \mu_{Al}$ and $\Delta \mu_{M}$ are the differences between the relative chemical potentials of Al and M atoms, respectively, and the chemical potentials of solid simple substance, which can be expressed as

(3) $$\begin{array}{l} \Delta \mu_{Al}=\mu_{Al}-\mu_{Al}^{bulk}\\ \Delta \mu_{M}=\mu_{M}-\mu_{M}^{bulk} \end{array}$$

Here, $\mu_{Al}^{bulk}$ and $\mu_{M}^{bulk}$ are the chemical potentials of the metals Al and M, respectively, that is, the single-atomic energies in the elemental state.

In order to avoid the precipitation of the solid elements Al and M, the chemical potential of each atom in the defect phase should be less than that of the solid elements, that is,

(4) $$\begin{array}{l} \Delta \mu_{Al}\leq 0\\ \Delta \mu_{M}\leq 0 \end{array}$$

From the phase diagram of each Al-M system, Al_3_M was in equilibrium with the adjacent pure Al phase, as the non-stoichiometric ratio was Al-rich due to point defects. When the non-stoichiometric ratio was M-rich due to point defects, the Al_3_M phase was in equilibrium with the adjacent stoichiometric Al_2_M phase. In order to avoid the formation of the pure Al and Al_2_M secondary phase, the chemical potential should be limited by the following:

(5) $$\begin{array}{l} \Delta \mu_{Al}\leq 0\\ 2\Delta \mu_{Al}+\Delta \mu_{M}\leq \Delta H_{f}^{Al_{2}M} \end{array}$$

Here, $\Delta H_{f}^{Al_{2}M}$ is the formation enthalpy of the unit chemical formula Al_2_M in the solid state.

**Figure 1:**
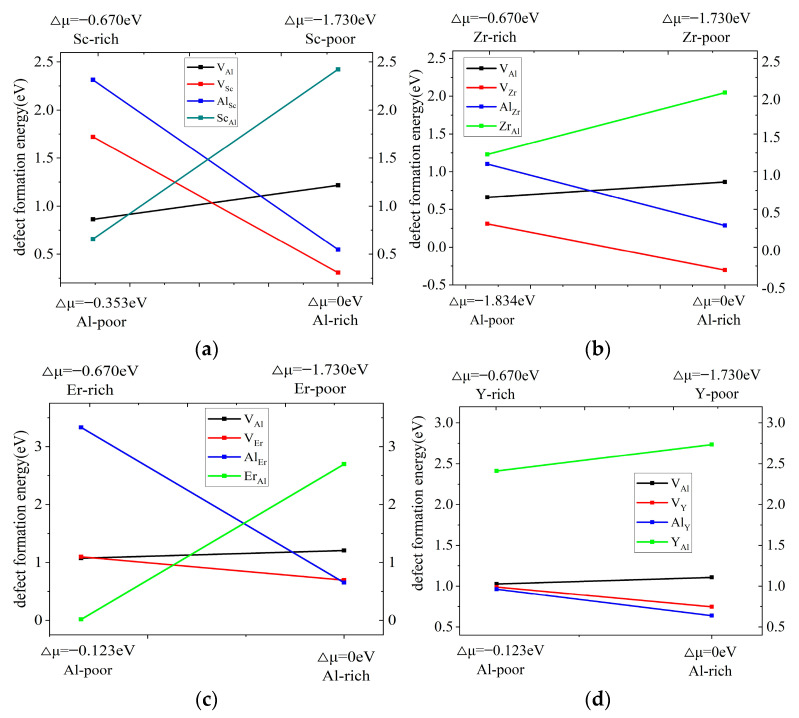
Formation energies of vacancy and antisite defects of L1_2_-Al_3_M phase: (**a**) Al_3_Sc defect; (**b**) Al_3_Zr defect; (**c**) Al_3_Er defect; and (**d**) Al_3_Y defect.

The formation energies of four kinds of defects in the L1_2_-Al_3_M phase were calculated, as shown in Figure 1. Under the Al-rich condition, the point defects of V_Sc_ obtained low formation energy and were the main point defects for the Al_3_Sc phase, and the point defects of Al_Er_ and V_Er_ were the main point defects for the Al_3_Er phase. The Sc_Al_ and Er_Al_ defects were the main point defects for the Al_3_Sc phase and Al_3_Er phase owing to the lowest formation energy under Sc-rich and Er-rich conditions. The point defects of the Al_3_Sc phase under Al-rich and Sc-rich conditions were well consistent with Ref. [23]. On the other hand, the change of the stoichiometric ratio had little effect on the formation energy of the point defects in the Al_3_Zr phase and Al_3_Y phase. The V_Zr_ and Al_Y_ defects obtained the lowest formation energy for the Al_3_Zr phase and Al_3_Y phase regardless of the Al-rich, Zr-rich, and Y-rich conditions. Furthermore, the defect energy of V_Zr_ was a negative value, indicating that the Al_3_Zr phase was inclined to form stable V_Zr_ vacancy defects under the Al-rich condition. The formation energy of the Y_Al_ antisite was always the highest; thus, it was difficult to form Y_Al_ antisite defects in the Al_3_Y phase. Shi et al. [23] suggested that the point defect formation energy was dependent on the electronic structure and the value of the electronic density of state (DOS) at the Fermi level. However, V_Al_ was the primary point defect near the stoichiometry [23]. Similar vacancy defects were reported in the Ni_3_Al phase [18], where vacancies defect on the Ni sublattice was the main point defect in the Ni_3_Al phase.

### 2.2. Vacancy-Mediated Atomic Migration

#### 2.2.1. Al Atomic Migration

**Figure 2:**
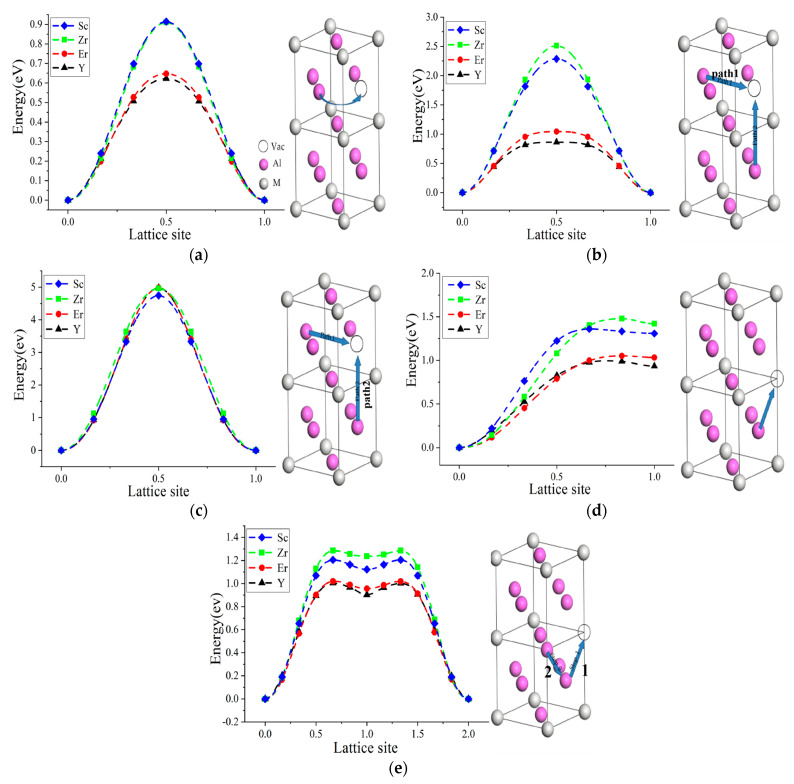
Energy profiles for Al atom diffusion in Al_3_M phase: (**a**) NNJ(Al-V_Al_); (**b**) NNNJ1(Al-V_Al_); (**c**) NNNJ2(Al-V_Al_); (**d**) NNJ(Al-V_M_); and (**e**) ASB(Al_M_-Al-V_M_). The blue arrow represented the path of atomic jump.

Figure 2a–c show the energy profiles for Al atomic diffusion mediated by V_Al_ in the Al_3_M phase. The nearest-neighbor site Al around V_Al_ can migrate to V_Al_ through the symmetrical NNJ pathways (denoted as NNJ(Al-V_Al_)), and the energy profile was symmetrical due to the restoration of the local disordered structures (Figure 2a). The highest energies of the energy profile corresponding to the migration barrier were 0.913 eV, 0.914 eV, 0.647 eV, and 0.622 eV for Al_3_Sc, Al_3_Zr, Al_3_Er, and Al_3_Y, respectively, indicating that NNJ(Al-V_Al_) with the low migration barrier was the preferred diffusion path for Al_3_M phases owing to the direct jump to V_Al_. Furthermore, the migration barrier of Al_3_Sc was almost the same as that of Al_3_Zr, while Al_3_Er and Al_3_Y obtained low migration barriers. The different migration barrier for the NNJ path can be attributed to the different M atomic sizes. The Er and Y atoms had large atomic radii; thus, the Al_3_Er and Al_3_Y obtained high lattice gaps, where Al atoms can migrate through the large atomic gaps to V_Al_, obtaining a lower migration barrier.

Al atoms that occupied the next-nearest-neighbor sites of V_Al_ can migrate to V_Al_ through two types of diffusion paths, denoted as NNNJ1 and NNNJ2, as shown in Figure 2b,c. As for the NNNJ1 path, the order of migration energy was Al_3_Zr > Al_3_Sc > Al_3_Er > Al_3_Y. Al atoms migrated through the quadrangle composed of the nearest-neighbor Al atoms, where the quadrangle gap became large with the increase in M atomic radius; correspondingly, the migration energy decreased with the increase in M atomic radius. However, the migration energies of the NNNJ2 path were almost the same and significantly increased owing to the high density of the quadrangle with two Al atoms and two M atoms. Compared with the NNJ migration, the Al atoms through the NNNJ1 and NNNJ2 paths needed to cross the quadrangle composed of four neighboring atoms, illustrating higher migration energy [23]. Therefore, the tendency of Al migration by NNNJ was very low.

The diffusion of Al atoms was also mediated by V_M_, including the NNJ and ASB migration paths. Figure 2d shows the migration-energy profile for the NNJ path of Al atom mediated by V_M_ (denoted as NNJ(Al-V_M_)), where the migration barriers mediated by V_M_ were still higher than that by V_Al_. Meanwhile, the final state of migration was unstable due to the local disorder by the migration of Al atoms to V_M_. In this sense, NNJ(Al-V_M_) was not the preferred migration path. As for the ASB migration (Figure 2e), Al_M_ occupied the next-nearest-neighbor site of V_M_, and the nearest-neighbor Al atoms migrated to V_M_, forming V_Al_ (step 1). Then, Al_M_ migrated to the newly formed V_Al_ (step 2), denoted as ASB(Al_M_-Al-V_M_). The order of migration energy for AS and ASB was similar to that for NNNJ1 as Al_3_Zr > Al_3_Sc > Al_3_Er > Al_3_Y, which can be explained by the different M atomic radii. Although the disorder of the migration final state was restored to the migration initial state, the ASB migration of Al atoms mediated by V_M_ was restricted due to the higher migration barrier of the NNJ migration by V_Al_. From the above discussion, the NNJ(Al-V_Al_) path had the lowest migration energy and was the main migration pathway for Al atomic migration.

#### 2.2.2. M Atomic Migration

**Figure 3:**
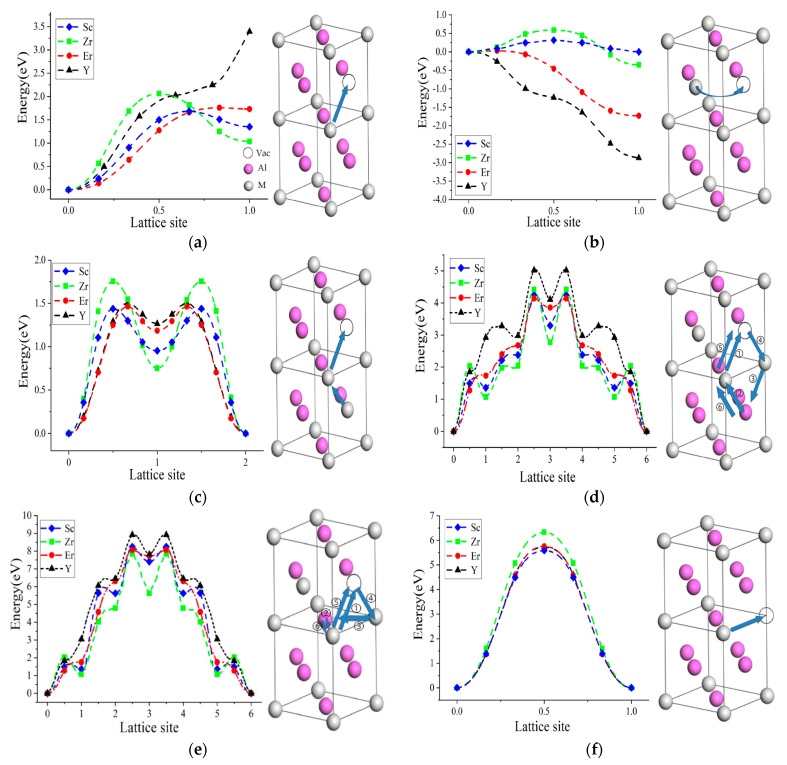
Energy profiles for M (M = Sc, Zr, Er, Y) atomic diffusion in Al_3_M phase: (**a**) NNJ(M-V_Al_); (**b**) AS(M_Al_-V_Al_); (**c**) ASB(M_Al_-M-V_Al_); (**d**) straight 6JC; (**e**) bent 6JC; and (**f**) NNNJ(M-V_M_). The blue arrow represented the path of atomic jump.

The migration of M atoms mediated by V_Al_ included the NNJ, AS, ASB, and 6JC paths. The migration of the nearest-neighbor M atoms jumped to V_Al_, denoted as NNJ(M-V_Al_), as shown in Figure 3a. The migration barriers of NNJ(M-V_Al_) were far higher than that of NNJ(Al-V_Al_). Meanwhile, the final state of migration was unstable due to the local disorder with the formation of M_Al_ and V_M_ defects. However, the Y atoms needed to overcome the increasing energy barrier during the migration process, suggesting that this migration path of Y atoms was energetically prohibited.

The AS migration path was another M diffusion path, where the M_Al_ atoms directly migrated to V_Al_ (denoted as AS(M_Al_-V_Al_)), as shown in Figure 3b. The migration barriers of the AS(M_Al_-V_Al_) path were very low compared with that of NNJ(M-V_Al_). Except for the Al_3_Sc phase, the final state energies of the Al_3_Zr, Al_3_Er, and Al_3_Y phases were negative, which was inconsistent with the fact that the local disorder restored to their initial state after M atomic migration. It suggested that the AS(M_Al_-V_Al_) path did not exist for the Al_3_Zr, Al_3_Er, and Al_3_Y phases in terms of energy. Furthermore, the AS(Sc_Al_-V_Al_) migration path was limited to some extent due to the difficulty of coexisting V_Al_ and Sc_Al_ in nearby locations.

For the ASB migration path, M_Al_ atoms and V_Al_ occupied the site of Al atoms (Figure 3c). The nearest-neighbor M firstly jumped to V_Al_, newly forming a V_M_ and M_Al_ atom; then, the M_Al_ atom migrated to the new V_M_ vacancy, resulting in a V_Al_ (denoted as ASB(M_Al_-M-V_Al_)). Obviously, the local disorder of the final state was consistent with that of the initial state; therefore, the migration-energy curve of the M atoms was symmetric. These two migration steps corresponded to the two saddle-curve characteristics of M atomic-migration energy. The Zr atomic migration by the ASB path in the Al_3_Zr phase obtained the maximum migration energy with 1.754 eV, which was lower than that of NNJ(M-V_Al_) with 2.059 eV. Meanwhile, the migration barriers of the Er, Y, and Sc atoms were nearly the same for the ASB path. However, the ASB pathway was limited due to the simultaneous presence of both M_Al_ and V_Al_ defects.

The 6JC path of M atomic migration mediated by V_Al_ consisted of the straight 6JC path and the bent 6JC path, as shown in Figure 3d,e. The six steps in the straight 6JC and bent 6JC were described in detail in Ref. [23]. The energy curves of M diffusion by straight 6JC and bent 6JC were symmetric due to the restoration of local disorder after the six-step migration process. The first step (M-V_Al_) in the straight 6JC and bent 6JC paths was similar to that of NNJ(M-V_Al_). In the first step, the migration energy of the Y atom increased during the migration process, while the migration energy of the Sc and Zr elements decreased after the migration to V_Al_. Thus, the Y atomic diffusion obtained far higher migration energy than the Er, Sc, and Zr atoms. The high migration barriers for the Y, Er, Sc, and Zr atoms indicated the straight 6JC and bent 6JC paths were energetically restricted.

The migration of M atoms mediated by V_M_ is also shown in Figure 3f. The M atom at the next-nearest-neighbor site jumped to V_M_ (denoted as NNNJ(M-V_M_)), and the energy profile of the M diffusion was symmetric owing to the restoration of local disorder. The Zr atom obtained the higher migration barrier than that of the Er, Sc, and Y atoms due to the dense structure of the Al_3_Zr phase. However, the Sc atomic diffusion had the lowest migration barrier, which did not agree with the effect of atomic size. Therefore, the migration barrier was related not only to the atomic radius of M but also to the electronic structure of the M atom [23]. Furthermore, the migration barriers of M atoms for NNNJ(M-V_M_) were much higher than that for NNJ(M-V_Al_) and ASB(M_Al_-M-V_Al_), suggesting that the NNNJ(M-V_M_) migration path was not a preferred migration path.

From the above discussion, except the AS(Sc_Al_-V_Al_) path being the preferred path for Sc atomic migration, the NNJ(M-V_Al_) and ASB(M_Al_-M-V_Al_) paths contributed to M atomic migration, while the straight 6JC, bent 6JC, and NNNJ(M-V_M_) paths were energetically prohibited.

### 2.3. Diffusion Activation Barrier

In the process of vacancy-mediated atomic diffusion, the activation barrier had a decisive influence on atomic diffusion and can be expressed as [23]:

(6) $$\Delta E_{a}=\Delta E_{f}^{defect}+\Delta E_{m}$$

Here, $\Delta E_{{}_{f}}^{defect}$ is the defect formation energy of the initial state, and $\Delta E_{m}$ is the migration barrier for different migration paths.

Table 1 shows that the calculated diffusion activation energies of the Al and M atoms in the Al_3_M phases. The calculation of the Al_3_Sc phase generally agreed with that of Ref. [23]. Under the Al-rich condition, the Al atomic-diffusion activation barriers of NNJ(Al-V_M_) were lower than that of NNJ(Al-V_Al_) in the Al_3_M phases, which was attributed to the low V_M_ formation energies under the Al-rich condition. However, the diffusion path of NNJ(Al-V_M_) was restricted due to the unstable final state (Figure 2d). Thus, NNJ(Al-V_Al_) diffusion was the main diffusion path for Al atoms under both Al-rich and M-rich conditions.

For M atomic diffusion, NNJ(M-V_Al_) diffusion obtained the low activation barriers for the Sc, Zr, Er, and Y atoms under both Al-rich and M-rich conditions, thus becoming the energetically preferred diffusion path. The diffusion activation barriers of Y atoms were far higher than that of the Sc, Zr, and Er elements due to their large atomic radii. Although the diffusion barriers of AS(Sc_Al_-V_Al_) and ASB(Er_Al_-Er-V_Al_) were much lower under the M-rich condition, their contribution to Sc atomic and Er atomic diffusions was limited due to the difficulty of coexisting V_Al_ and Sc_Al_, Er_Al_ defects in nearby locations.

The activation barriers of Al atomic and M atomic diffusions mediated by V_Al_ under the M-rich condition were generally lower than that under the Al-rich condition owing to the lower formation energy of V_Al_. The diffusion activation barriers of Al atoms in the Al_3_M phase were lower than that of M atoms. Moreover, ASB, NNNJ, straight 6JC, and bent 6JC mechanisms with high diffusion activation barriers were not factually executed for Al atomic and M atomic diffusions under both Al-rich and M-rich conditions. Therefore, the NNJ(Al-V_Al_) diffusion under the M-rich condition was the most preferred diffusion mechanism in the Al_3_M phase, and the order of activation barriers was Al_3_Zr < Al_3_Y < Al_3_Er < Al_3_Sc. It should be noted that these calculations were Al-atomic-self-diffusion activation barriers in the Al_3_M phase. It implied that the Al_3_Sc phase had high stability with a high self-diffusion activation energy, while the Al_3_Zr phase was relatively unstable with a low self-diffusion activation barrier, which well agreed with the fact that the Al_3_Zr phase transformed from the L1_2_ structure to D0_23_ structure at high temperatures [26].

### 2.4. Atomic Diffusion of Core-Shelled L1_2_-Al_3_(N,Zr)

The addition of the Er, Y, Sc, and Zr elements in aluminum alloys typically formed L1_2_-Al_3_M phases with a core-shelled structure. Due to the low interface energy between the Al_3_Zr phase and aluminum matrix, the Al_3_Zr phase tended to form a shell layer, and the Al_3_N (N = Sc, Er, Y) phase was inclined to form a core layer, where the core-shell structure was denoted as Al_3_(N,Zr). Atomic diffusion between the Al_3_N core and Al_3_Zr shell was investigated in this section. The Zr atomic diffusion to the Al_3_N core and the N atomic diffusion to the Al_3_Zr shell were respectively calculated. The previous investigation showed that Zr atoms in the Al_3_Zr shell reciprocally substituted the site of N atoms in the Al_3_N core [27].

As described in Section 2.3, NNJ(M-V_Al_) was the preferred diffusion path for Zr atoms in the Al_3_N core, where Zr atoms occupied the site of N atoms at the nearest-neighbor site of V_Al_. The formation energy of V_Al_ was affected by Zr atomic substitution and can be expressed as

(7) $$E_{f}^{def}(Zr_{N}^{})=E_{total}^{def}(Zr_{N})-E_{total}^{bulk}-\sum_{i}\Delta n_{i}\mu_{i}-\mu_{Zr}$$

Here, $E_{total}^{def}(Zr_{N})$ is the total energy of defective supercell with Zr substitution. μ_Zr_ is the chemical potential of Zr atoms.

For the N atomic substitution for Zr atoms in the Al_3_Zr shell, the formation energies of V_Al_ defect with N atomic substitution are expressed as

(8) $$E_{f}^{def}(N_{Zr})=E_{total}^{def}(N_{Zr})-E_{total}^{bulk}-\sum_{i}\Delta n_{i}\mu_{i}-\mu_{N}$$

Here, $E_{total}^{def}(N_{Zr})$ is the total energy of the defective supercell with N substitution. μ_N_ is the chemical potential of N atoms.

Table 2 shows that the formation energy of the V_Al_ defect with Zr substitution was within that of the pure Al_3_N phase between Al-rich and N-rich, and Zr substitution had little influence on the formation energy of the V_Al_ defect due to the atomic radius of Al being close to that of Zr. As shown in Table 3, the Sc substitution for Zr atoms slightly increased the formation energy of the V_Al_ defect, while the Er and Y substitutions significantly increased the formation energy of the V_Al_ defect.

**Figure 4:**
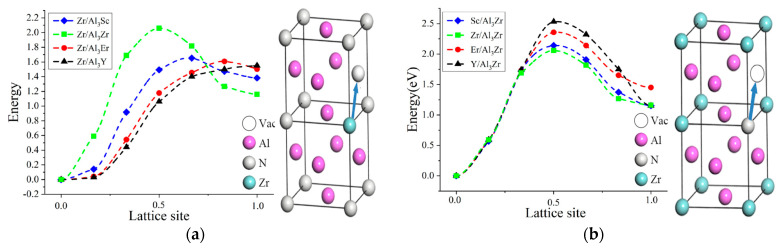
Atomic-migration energy of core-shelled L1_2_-Al_3_Zr(N): (**a**) the migration of Zr atom of Al_3_Zr shell layer to core Al_3_N phase and (**b**) the migration of N atom of Al_3_N core layer to shell Al_3_Zr phase. The blue arrow represented the path of atomic jump.

Zr atoms were considered to migrate through the NNJ path mediated by V_Al_ in the Al_3_N core, as shown in Figure 4a. The migration barriers of Zr diffusion in the Al_3_Sc, Al_3_Er, and Al_3_Y cores were 1.658 eV, 1.653 eV, and 1.570 eV, respectively, which were far lower than that in the Al_3_Zr phase with 2.059 eV. The order of migration barriers for Zr atoms in the Al_3_N core was Al_3_Sc > Al_3_Er >Al_3_Y. With the increase in the N atomic radius for the Al_3_N core, Zr atoms easily migrated due to the increase in the lattice gap. Thus, Zr atomic diffusion in the Al_3_Y core obtained the lowest migration energy. Figure 4b illustrates that the diffusion of Sc, Er, and Y atoms in the Al_3_Zr shell obtained migration barriers with 2.14 eV, 2.356 eV, and 2.53 eV, respectively, which were higher than that of Zr migration in the Al_3_Zr shell with 2.059 eV. The atomic-migration energy in the Al_3_Zr shell was sequentially Y > Er > Sc, which can be attributed to the high diffusion resistance for the large atomic radius.

As the diffusion activation barrier included vacancy formation energy and atomic-migration energy, the diffusion activation energies of Zr atoms in the Al_3_Sc, Al_3_Er, and Al_3_Y cores were 2.852 eV, 2.84 eV, and 2.633 eV, respectively. Additionally, the diffusion activation barriers of the Sc, Er, and Y atoms in the Al_3_Zr shell were 3.024 eV, 3.492 eV, and 3.889 eV, respectively. Compared with the diffusions of the Sc, Er, and Y atoms into the Al_3_Zr shell, Zr atoms were more inclined to enter the A_l3_Sc, Al_3_Er, and Al_3_Y cores based on diffusion activation energy. Furthermore, Zr atoms preferred to diffuse into the Al_3_Y core, while the diffusion of Zr atoms into the Al_3_Er and Al_3_Sc cores required higher activation barriers. It revealed that Zr atoms in the Al_3_Zr shell were inclined to diffuse into the Al_3_Y core during the subsequent aging process, thus resulting in no typical core-shelled structure. However, Zr atoms were difficult to diffuse into Al_3_Sc and Al_3_Er cores due to their high diffusion activation barrier, thus maintaining a typical core-shelled structure.

## 3. Computational Methods

Based on density functional theory (DFT) [28], first-principles calculations were carried out by Vienna ab initio simulation package (VASP) software [29]. The projector augmented wave (PAW) with the Perdew–Burke–Ernzerh (PBE) method of generalized gradient approximation (GGA) was used to describe the exchange-correlation energy functional between electrons [30]. The electron configuration was described by the Al-3s^2^3p^1^, Sc-3s^2^3p^6^4s^1^3d^2^, Zr-4s^2^4p^6^5s^1^4d^3^, Er-6s^2^5p^6^5d^1^, and Y-4s^2^4p^6^5s^1^4d^2^ valence states, respectively. The kinetic energy cutoff of the plane-wave basis and the size of the k-mesh for the Brillouin zone were tested for self-consistent convergence. The geometric structure was optimized by the Monkhorst-Pack k-point grids with linear k-mesh analytical values of less than 0.032π/Å. The total energy was calculated using the linear tetrahedron method with the Blöchl correction when the total energy converged to 10^−4^ eV/atom. The lattice constants (a_0_) were predicted as 4.042 Å, 4.103 Å, 4.108 Å, and 4.232 Å for fcc-Al, L1_2_-Al_3_Sc, L1_2_-Al_3_Zr, and L1_2_-Al_3_Er, respectively, which were well consistent with Ref. [31].

There were two sublattices in the L1_2_-Al_3_M (M = Sc, Zr, Er, Y) unit cell, the Al sublattice located at the 3c position (0,0.5,0.5) and the M sublattice located at the 1a (position (0,0,0). Therefore, there were four types of primary point defects in Al_3_M, including Al vacancy (V_Al_), M vacancy (V_M_), Al antisite (Al_M_), and M antisite (M_Al_). In order to reduce vacancy density and limit the interaction between defects, 108 atoms in a 3 × 3 × 3 supercell were used in this calculation.

The diffusion mechanism of the L1_2_-Al_3_M phase mediated by vacancy included from the nearest-neighbor to complex hopping sequences, such as nearest-neighbor jump (NNJ), next-nearest-neighbor jump (NNNJ), antistructural sublattice (AS), antistructural bridge (ASB), and 6-jump cycle (6JC) [23]. The CI-NEB method [24] was used to calculate the energy profile. A series of atomic positions were inserted between the initial and final states to construct the model, and then, each insertion point model was relaxed until the force threshold at the insertion point was 10^−2^ eV/Å. By this method, the vacancy diffusion behavior of the L1_2_-Al_3_M phase was comparatively studied, and the atomic-diffusion interaction between the core layer and the shell layer for the core-shelled L1_2_-Al_3_M phase was also discussed.

## 4. Conclusions

Atomic-diffusion mechanisms in L1_2_-Al_3_M (M = Sc, Er, Y, Zr) phases were investigated based on a first-principles calculation. The main conclusions are summarized as follows:

(1) NNJ(Al-V_Al_) and NNJ(M-V_Al_) diffusions were the energetically preferred diffusion paths under both Al-rich and M-rich conditions. The straight 6JC, bent 6JC, and NNNJ were significantly inhibited owing to their high activation barriers. Other diffusion paths, such as NNJ(Al-V_M_), AS(Sc_Al_-V_Al_), and ASB(Er_Al_-Er-V_Al_), were limited due to the unstable final-state structure and the difficulty of coexisting V_Al_ and M_Al_ defects.
(2) The order of activation barriers for NNJ(Al-V_Al_) was Al_3_Zr < Al_3_Y < Al_3_Er < Al_3_Sc. The Al_3_Sc phase had high stability with a high self-diffusion activation barrier, while the Al_3_Zr phase was relatively unstable with a low self-diffusion activation energy.
(3) Compared with the diffusion of the Sc, Er, and Y atoms in the Al_3_Zr shell, Zr atoms were more inclined to diffuse into the Al_3_Y, Al_3_Er, and Al_3_Sc cores, and the activation barriers were as follows: Al_3_Y < Al_3_Er < Al_3_Sc. Thus, Zr atoms were prone to diffuse into the Al_3_Y core, resulting in no core-shelled structure.

## Figures and Tables

**Table 1:** Diffusion activation barrier of L1_2_-Al_3_M.

System	Diffusion Atoms	Diffusion Mechanisms	Activation Barrier (eV)	
			Al-Rich	Sc-Rich
Al_3_Sc	Al	NNJ(Al-V_Al_)	2.131–2.250 [23]	1.778–1.689 [23]
		NNJ(Al-V_Sc_)	1.668–2.352 [23]	3.081–3.916 [23]
		NNNJ1(Al-V_Al_)	3.503–3.825 [23]	3.150–3.244 [23]
		NNNJ2(Al-V_Al_)	5.963–6.441 [23]	5.609–5.860 [23]
		ASB(Al_Sc_-Al-V_Sc_)	2.346–2.628 [23]	5.574–6.297 [23]
	Sc	NNJ(Sc-V_Al_)	2.900–3.124 [23]	2.547–2.543 [23]
		AS(Sc_Al_-V_Al_)	3.956–4.144 [23]	1.178–1.148 [23]
		ASB(Sc_Al_-Sc-V_Al_)	5.079–5.546 [23]	2.958–2.640 [23]
		NNNJ(Sc-V_Sc_)	5.893–8.881 [23]	7.306–10.625 [23]
		S6JC(Sc----V_Al_)	5.489–4.759 [23]	5.135–6.504 [23]
		B6JC(Sc----V_Al_)	9.430–10.154 [23]	9.076–10.734 [23]
Al_3_Zr	Al	NNJ(Al-V_Al_)	1.776	1.572
		NNJ(Al-V_Zr_)	1.175	1.788
		NNNJ1(Al-V_Al_)	3.376	3.172
		NNNJ2(Al-V_Al_)	6.183	5.830
		ASB(Al_Zr_-Al-V_Zr_)	1.736	3.166
	Zr	NNJ(Zr-V_Al_)	2.926	2.721
		AS(Zr_Al_-V_Al_)	-	-
		ASB(Zr_Al_-Zr-V_Al_)	4.666	3.645
		NNNJ(Zr-V_Zr_)	6.029	6.642
		S6JC(Zr----V_Al_)	5.373	5.169
		B6JC(Zr----V_Al_)	8.755	8.550
Al_3_Er	Al	NNJ(Al-V_Al_)	1.856	1.722
		NNJ(Al-V_Er_)	1.748	2.149
		NNNJ1(Al-V_Al_)	1.969	1.888
		NNNJ2(Al-V_Al_)	6.187	6.053
		ASB(Al_Er_-Al-V_Er_)	2.849	5.932
	Er	NNJ(Er-V_Al_)	2.966	2.832
		AS(Er_Al_-V_Al_)	-	-
		ASB(Er_Al_-Er-V_Al_)	5.370	2.555
		NNNJ(Er-V_Zr_)	6.451	6.853
		S6JC(Er----V_Al_)	5.350	5.216
		B6JC(Er----V_Al_)	9.220	9.086
Al_3_Y	Al	NNJ(Al-V_Al_)	1.730	1.649
		NNJ(Al-V_Y_)	1.725	1.968
		NNNJ1(Al-V_Al_)	1.969	1.888
		NNNJ2(Al-V_Al_)	6.090	6.009
		ASB(Al_Y_-Al-V_Y_)	2.885	3.452
	Y	NNJ(Y-V_Al_)	4.507	4.426
		AS(Y_Al_-V_Al_)	-	-
		ASB(Y_Al_-Y-V_Al_)	5.347	4.942
		NNNJ(Y-V_Sc_)	6.472	6.715
		S6JC(Y----V_Al_)	6.203	6.172
		B6JC(Y----V_Al_)	10.230	10.149

**Table 2:** Formation energy of V_Al_ defect in pure and Zr-substituted Al_3_N core.

	Formation Energy of V_Al_ (eV)		
	Al_3_Sc	Al_3_Er	Al_3_Y
Pure	0.863–1.217	1.074–1.208	1.026–1.107
Zr substitution	1.194	1.187	1.063

**Table 3:** Formation energy of V_Al_ defect in pure and N-substituted Al_3_Zr shell.

	Pure	Sc	Er	Y
V_Al_ (eV)	0.661–0.865	0.884	1.136	1.359

## Data Availability

The data presented in this study are available on request from the corresponding author.
